# Endogenous expression and localization of CAV-1::GFP in *C. elegans*

**DOI:** 10.17912/micropub.biology.000311

**Published:** 2020-09-21

**Authors:** Dillon E. Sloan, Joshua N. Bembenek

**Affiliations:** 1 University of Michigan

**Figure 1. Endogenous CAV-1::GFP:**
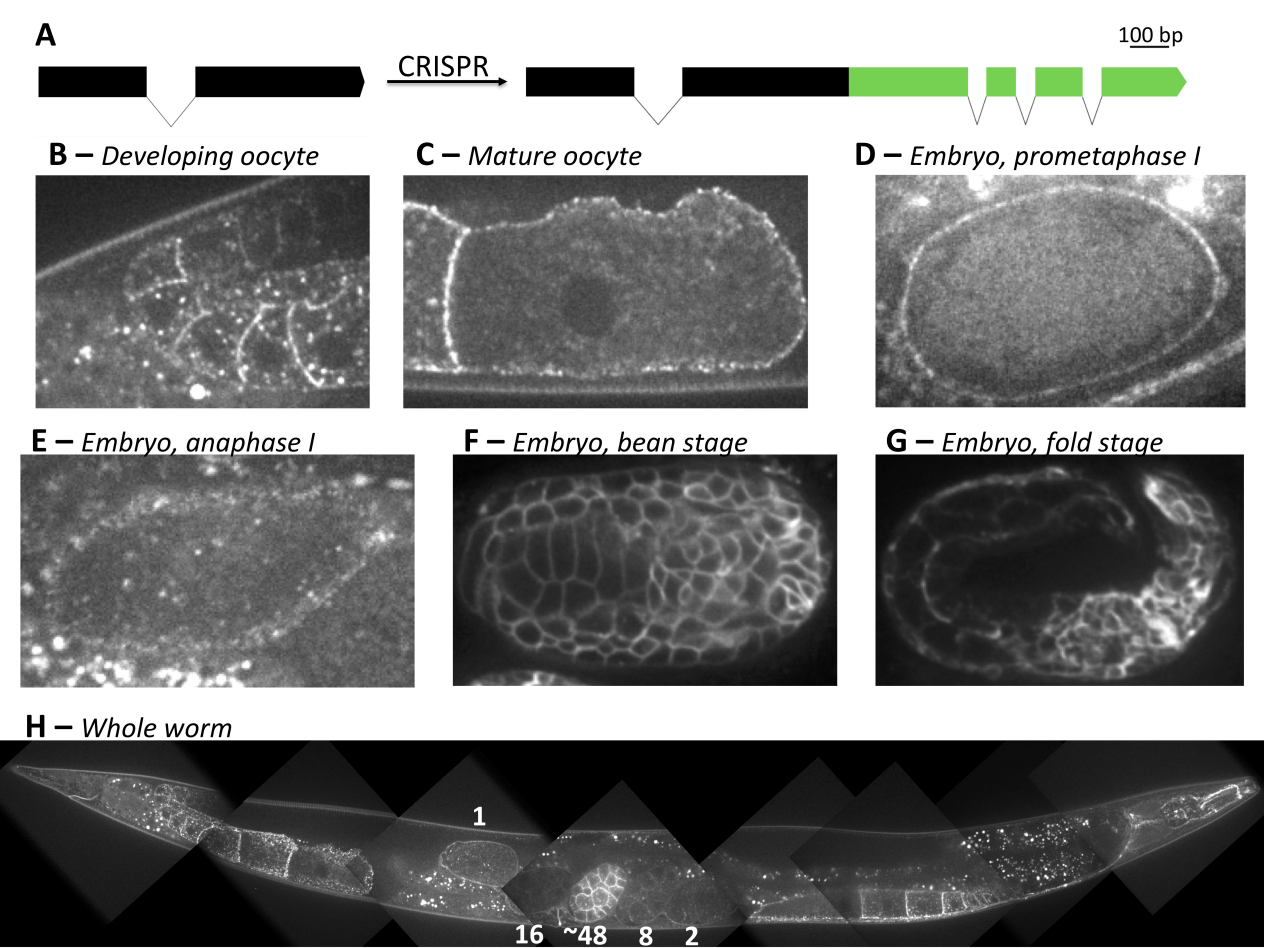
(A) Schematic of *cav-1* edit (B-G) Single-plane images of CAV-1::GFP localization: at the bend of the gonad arm (B), in the -1 oocyte (C), in a prometaphase I embryo (D), in an anaphase I embryo (E), in a bean stage embryo (F), and in a fold-stage embryo (G). (H) CAV-1::GFP in adult worm. Numbers correspond with number of cells in embryo.

## Description

Caveolins are integral membrane proteins responsible for the formation of caveolae, invaginations of the plasma membrane linked to various disease states (Parton *et al.* 2020). In *C. elegans,* there are two caveolin proteins, CAV-1 and CAV-2. The *cav-1* gene shares homology with all three mammalian caveolin genes (Tang *et al.* 1997). *C. elegans* CAV-1 protein does not appear to form caveolae, but a double-knockout mutant of *cav-1* and *cav-2* affects egg laying, and knockdown of *cav-1* affects locomotion in a dynamin mutant background (Parker *et al.*. 2007, Kirkham *et al.* 2008, Sato *et al.* 2008). Based on exogenous expression, CAV-1::GFP is known to localize to cortical granules and the plasma membrane in oocytes and the early embryo, to the plasma membrane in later embryos, as well as to the neuromuscular system in larvae and adult worms (Sato *et al.* 2006, Bembenek *et al.* 2007, Parker *et al.* 2007).

Using CRISPR/Cas9, we generated an endogenous CAV-1::GFP fluorescent fusion strain, primarily to create a cortical granule marker (Figure 1A). Imaging endogenous CAV-1::GFP revealed similar patterns as previously reported in the germ line (Sato *et al.* 2006). However, unlike the *pie-1* driven exogenous line, endogenous CAV-1::GFP was not clearly detected in the distal gonad until the bend region of the gonad arm, where it localized to the plasma membrane, and vesicle-shaped structures (Figure 1B). Oocytes had slightly higher expression showing enrichment at plasma membrane localization but faint cytoplasmic signal, and no obvious enrichment to vesicles (Figure 1C). After ovulation, CAV-1::GFP remained on the plasma membrane during meiosis I and was no longer detected by meiosis II (Figure 1D,E). In 5/5 anaphase I embryos, CAV-1::GFP was not significantly localized to cortical granules at a time when separase could be observed on vesicles. Overall, endogenous CAV-1::GFP signal is less intense in the germline than exogenous *pie-1*-drivenlines. However, endogenous CAV-1::GFP signal became increasingly intense at the plasma membrane sometime after the 16-cell embryo stage (Figure 1H). The membrane signal remains intense through later stages of embryonic development (Figure 1F,G). This potentially indicates a dynamic role for *cav-1* in embryonic development. Finally, adult somatic expression appeared to follow previously observed patterns (Figure 1H, Parker *et al.* 2007).

In summary, we have developed an endogenous CAV-1::GFP line by CRISPR, which can be of use to researchers interested in CAV-1.

## Methods

CRISPR/Cas9 Gene editing

We followed the CRISPR/Cas9 protocol generated by Seydoux lab for C-terminal GFP tagging of the C. elegans *cav-1* gene (Figure 1A, Paix *et al.* 2015). The repair template was amplified from the pDD282 plasmid. All guide RNAs and oligos were obtained commercially.

The primer sequences are listed below:

*cav-1*::GFP Forward *(termed DS_027)*:

ACGGAATCAATCAAGAAACTACTGCTCCATGCGTCATGAGTAAAGGAGAAGAATTGTTCACTG

*cav-1*::GFP Reverse *(termed DS_026)*:

AGTAAAATGAATTTGAGATAAATTAAATAAATTTACTTGTAGAGCTCGTCCATTCC

The crRNA sequence recognizing the spacer upstream of NGG is as follows:

*(Termed DS_g003):* AUUAAAUAAAUUUAGACGCA

Microscopy

Imaging was done as indicated in Bai *et al.* 2020. Embryos were staged using the localization of separase fused with a red fluorescent protein as well as embryo position and age in the animal.

## Reagents

JAB192: *cav-1*(*erb78*[*cav-1*::GFP])

This strain is available upon request.
